# Identification of Domains and Factors Involved in MINIYO Nuclear Import

**DOI:** 10.3389/fpls.2019.01044

**Published:** 2019-09-05

**Authors:** Ramon Contreras, Paraskevi Kallemi, Mary Paz González-García, Aleksandra Lazarova, José Juan Sánchez-Serrano, Maite Sanmartín, Enrique Rojo

**Affiliations:** Centro Nacional de Biotecnología–CSIC, Cantoblanco, Madrid, Spain

**Keywords:** stem cell, differentiation, nuclear localization signal, importin, GTPase

## Abstract

The transition of stem cells from self-renewal into differentiation is tightly regulated to assure proper development of the organism. *Arabidopsis MINIYO* (*IYO*) and its mammalian orthologue *RNA polymerase II associated protein 1* (*RPAP1*) are essential factors for initiating stem cell differentiation in plants and animals. Moreover, there is evidence suggesting that the translocation of IYO and RPAP1 from the cytosol into the nucleus functions as a molecular switch to initiate this cell fate transition. Identifying the determinants of IYO subcellular localization would allow testing if, indeed, nuclear IYO migration triggers cell differentiation and could provide tools to control this crucial developmental transition. Through transient and stable expression assays in *Nicotiana benthamiana* and *Arabidopsis thaliana*, we demonstrate that IYO contains two nuclear localization signals (NLSs), located at the N- and C-terminus of the protein, which mediate the interaction with the NLS-receptor IMPA4 and the import of the protein into the nucleus. Interestingly, IYO also interacts with GPN GTPases, which are involved in selective nuclear import of RNA polymerase II. This interaction is prevented when the G1 motif in GPN1 is mutated, suggesting that IYO binds specifically to the nucleotide-bound form of GPN1. In contrast, deleting the NLSs in IYO does not prevent the interaction with GPN1, but it interferes with import of GPN1 into the nucleus, indicating that IYO and GPN1 are co-transported as a complex that requires the IYO NLSs for import. This work unveils key domains and factors involved in IYO nuclear import, which may prove instrumental to determine how IYO and RPAP1 control stem cell differentiation.

## Introduction

*Arabidopsis* MINIYO (IYO) is a key pro-differentiation factor whose migration from the cytosol into the nucleus coincides with the onset of stem cell differentiation (Sanmartin et al., 2011; Sanmartin et al., 2012; Muñoz et al., 2017). Identifying the domains and mechanisms that regulate IYO migration into the nucleus is thus crucial to elucidate where and how IYO functions to activate cell differentiation. Nuclear protein import requires in many cases active transport mechanisms, which are dependent on the presence of a specific nuclear localization signals (NLS) in the sequence of the proteins to be imported (Dingwall and Laskey, 1991). Classical NLSs are rich in basic amino acids (aa) and can be classified as monopartite or bipartite. Monopartite NLSs have a consensus (K(K/R)X(K/R) sequence, which is not only conserved but also essential for functionality (Lange et al., 2007). On the other hand, bipartite NLSs include two regions rich in lysine and other basic aa separated by a spacer of 10 to 12 aa (Lange et al., 2007). In addition, there is a third type of non-classical NLS, found only in yeast and plant proteins, whose consensus sequence (KIPIK) is also rich in lysine (Hicks et al., 1995). Canonical transport into the nucleus mediated by NLS-type signals occurs in a two-step process: in the cytoplasm, proteins with an NLS interact through this domain with the importin-α subunit of nuclear import receptors. In turn, the importin-β subunit of the receptor interacts with nucleoporins to facilitate transport of the receptor-cargo complex across the nuclear pore (Smith et al., 1997; Macara, 2001). Once the heterotrimer is inside the nucleus, active GTP-bound small GTPases of the RAN family (RAN-GTP) bind to the importins, dissociating the complex and releasing the cargo into the nucleus (Quimby and Dasso, 2003). Nuclear transport rate is dependent on the affinity of importins for the cargo NLS and on the importin availability (Dingwall and Laskey, 1991; Riddick and Macara, 2005).

IYO is orthologous to mammalian RPAP1 (Lynch et al., 2018), one of four RNA polymerase II associated proteins (RPAPs), which are present in all eukaryotes. In yeast and animal cells, RPAPs constitute a network of cross-interacting factors that participate in the cytosolic assembly of the RNA polymerase II (Pol II) macro-complex and in its nuclear import (Jeronimo et al., 2007; Gibney et al., 2008; Forget et al., 2010; Forget et al., 2013). In *Arabidopsis*, IYO associates with the RPAP2 orthologue RIMA and the RPAP4 orthologue GPN1 (Muñoz et al., 2017; Li et al., 2018), indicating that the RPAP interaction network is conserved in plants. Interestingly, disrupting *RIMA* function leads to an increase in the cytosolic/nuclear ratio of IYO, while IYO overexpression increases RIMA nuclear levels (Muñoz et al., 2017), suggesting that these plant RPAPs cooperate for import into the nucleus.

GPN1 belongs to the small family of GPN-loop GTPases (GPNs), composed of three members in eukaryotes. Many reports in yeast and animals link GPNs to nuclear protein transport (Forget et al., 2010; Calera et al., 2011; Carre and Shiekhattar, 2011; Minaker et al., 2013; Staresincic et al., 2011; Zeng et al., 2018). GPNs shuttle between the cytoplasm and the nucleus (Forget et al., 2010; Reyes-Pardo et al., 2012) and are structurally related to RAN GTPases, which are core components of the canonical nuclear transport cycle. In yeast, the GPN1 orthologue NPA3 interacts with the nucleoporin Nup133 and with the nuclear export receptor Crm1/Xpo1 (Staresincic et al., 2011), supporting a direct participation of GPN1 in transport across the nuclear pore. Depletion of mammalian GPN1 or GPN3 and of yeast NPA3 or GPN2 lead to increased cytoplasmic/nuclear ratio of Pol II (Forget et al., 2010; Calera et al., 2011; Staresincic et al., 2011; Minaker et al., 2013; Zeng et al., 2018). Moreover, GTPase dead versions of GPN1 dominantly interfere with Pol II nuclear accumulation (Carre and Shiekhattar, 2011). GPNs contain the five highly conserved G domains, G1 to G5, required for nucleotide binding and GTP hydrolysis in all GTPases (Bourne et al., 1991; Niesser et al., 2015) and are characterized by a Gly-Pro-Asn sequence (GPN motif) inserted into the GTPase core fold (Gras et al., 2007). Mutations in the GPN motif or in the G1 domain block GTPase activity in GPN1 and expression of these mutant versions cause Pol II accumulation in the cytoplasm (Carre and Shiekhattar, 2011; Forget et al., 2010; Niesser et al., 2015). The strong evidence for a connection of GPNs, and GPN1 in particular, with selective nuclear transport implies that studying the interaction of IYO with GPNs may be crucial to elucidate how IYO nuclear migration is achieved and how its pro-differentiation activity is regulated.

## Materials and Methods

### Plant Material and Growth Conditions

*Arabidopsis thaliana* Col-0 plants were grown in a soil/vermiculite mixture (3:1) under a long day photoperiod regime (16 h light/8 h dark) at 21°C, 60% relative humidity in a growth chamber. *Nicotiana benthamiana* plants were grown on 100% soil substrate in the greenhouse at 22°C day/19°C night. *35S::IYO-GFP*, *35S::IYO_ATG978_-GFP*, *35S::IYO_978REV_-GFP*, and *35S::IYO*_ΔNLSAB_*-GFP* were stably transformed in Col-0 plants.

### Constructs

Constructs were amplified with Phusion^®^ High-Fidelity polymerase (Thermo Fisher Scientific), cloned in the pDONR207 entry vector with the GATEWAY^®^ Cloning Technology System (Thermo Fisher Scientific; **Supplementary Table 1**) and sequence-verified. The inserts were transferred to the binary vectors pGWB5, pGWB6 (Nakagawa et al., 2007), pPZP-221 (provided by Dr. S. Prat), and pBIFC (donated by Dr. F. Parcy) for plant expression. To generate IYO deletion constructs, we used specific primers flanking the sequences to be deleted (NLSA, NLSB; **Supplementary Table 1**) followed by ligation with the T4 DNA ligase kit (Thermo Fisher Scientific). For the generation of the GPN1 mutated versions, GPN1_mGPN_ (GPN1_GPN108AAA_) and GPN1_mG1_ (GPN1_GSGK51AAAA_) directed mutagenesis was carried out following the specifications of the Q5 Site-Directed Mutagenesis Kit (New England Biolabs) using the primers suggested by the online application NEBaseChanger (**Supplementary Table 1**).

### Transient Expression in Nicotiana benthamiana

*N. benthamiana* leaves were infiltrated with *Agrobacterium* cultures and analyzed two days after infiltration by light microscopy using a Leica TCS SP5 laser scanning confocal microscope (Leica Microsystems). For the visualization of GFP and YFP, the samples were excited with the 488-nm laser, and emitted light in the 496–538 nm range was captured. For the visualization of RFP, the samples were excited with the 561-nm laser, and emitted light in the 570–630 nm range was captured. Single confocal optical sections are shown in all the figures, except in **Supplementary Figure 5**. Samples with two fluorophores were captured by sequential scanning to avoid signal bleed-through. For leptomycin B (LMB) treatments, 0.9 µM LMB was infiltrated in *N. benthamiana* leaves before imaging by confocal microscopy.

### Co-Immunoprecipitation

Plant material was ground in extraction buffer [50 mM Tris-HCl, pH 7.5; 150 mM NaCl; 2 mM DTT; 1 mM PMSF and 1X protease inhibitors (Roche)] and centrifuged at 20,000 g for 10 min at 4ºC. GFP-Trap Agarose beads (Chromotek) were equilibrated in extraction buffer supplemented with 0.5% TX-100 and 2% BSA and incubated with the samples at 4ºC for 3 h. The beads were washed four times with extraction buffer supplemented with 0.5% TX-100 and resuspended in 3X SDS-sample buffer for western blot analysis.

### Yeast Two-Hybrid Assays

GPN1, GPN3, and IYO full length, mutated, or truncated versions were cloned in the destination vector pGBKT7-GW (Gal4 DNA-binding domain, BD) or pGADT7-GW (Gal4 activation domain, AD). To assess protein interactions, the corresponding plasmids were co-transformed into *Saccharomyces cerevisiae* AH109 cells following standard heat-shock protocols. The empty vector pGADT7-GW was also co-transformed with pGBKT7 constructs as a negative control.

### Bioinformatics Applications

NLStradamus (www.moseslab.csb.utoronto.ca/NLStradamus/) and NLS Mapper (nls-mapper.iab.keio.ac.jp/cgi-bin/NLS_Mapper_form.cgi) online programs were used to identify possible NLS sequences in the IYO/RPAP1 sequences. Clustal Omega program (www.ebi.ac.uk/Tools/msa/clustalo/) was used for the multiple sequence alignment, visualized by ESPript 3.0 program (Robert and Gouet, 2014, http://espript.ibcp.fr/ESPript/cgi-bin/ESPript.cgi).

### Accession Numbers

Sequence data from this article can be found in The Arabidopsis Information Resource (TAIR) and in Phytozome databases under the following accession numbers: *AtIYO*: AT4G38440, *GPN1*: AT4G21800, *GPN2*: AT5G22370, *GPN3*: AT4G12790, *IMPA3*: AT4G02150, *IMPA4*: AT1G09270, and *IMPA6*: AT1G02690, *Vitis vinifera VvIYO*: GSVIVG01023952001, *Oryza sativa*, *OsIYO*: LOC_Os06g37640, *Populus trichocarpa*, *PtIYO*: Potri.009G139900, *Physcomitrella patents PpIYO*: Pp3c6_25040, and *Marchantia polymorpha MpIYO*: Mapoly0006s0149.

## Results

### Identification of Domains Involved in the Nuclear Import of IYO

**Figure 1:**
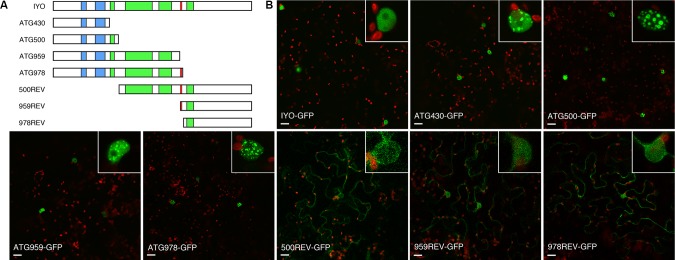
Characterization of IYO domains involved in nuclear/cytosolic distribution. **(A)** Schematic representation of IYO truncated versions that were fused to GFP, with RPAP1 domains (blue), ARM repeats (green), and the conserved RGG motif (red) highlighted. **(B)** Single confocal sections of *N. benthamiana* leaves transformed with the IYO-GFP translational constructs: IYO full length (IYO-GFP), IYO_ATG430_ (ATG430-GFP), IYO_ATG500_ (ATG500-GFP), IYO_ATG959_ (ATG959-GFP), IYO_ATG978_ (ATG978-GFP), IYO_500REV_ (500REV-GFP), IYO_959REV_ (959REV-GFP), and IYO_978REV_ (978REV-GFP). GFP signal is shown in green; chloroplasts signal in red. Insets show details of nuclei from each panel. Scale bar: 20 µm.

In previous work, we gained evidence suggesting that migration of IYO from the cytosol to the nucleus functions as a molecular switch to activate cell differentiation in *Arabidopsis* (Sanmartin et al., 2011; Sanmartin et al., 2012; Muñoz et al., 2017). However, the determinants of the subcellular location of IYO remained unknown. To find the domains responsible for IYO nuclear localization, we generated truncated versions of IYO fused to GFP. We designed the constructs according to predicted functional domains in the IYO sequence. IYO encodes a protein of 1,465 aa that contains conserved RPAP1_N and RPAP1_C domains (pfam08620 and pfam08621) located between aa 209–254 and 318–397, respectively; Armadillo repeats between aa 500–1,000; and the RGG motif, located between aa 959 and 961 (**Figure 1A**; **Supplementary Figure 1**). The RGG motif is important for the pro-differentiation activity of IYO (Sanmartin et al., 2011) and, therefore, could be involved in its nuclear localization. To characterize the relevance of these regions for IYO subcellular distribution, we generated the following truncated versions: constructs IYO_ATG430_, IYO_ATG500_, IYO_ATG959_, and IYO_ATG978_, which encode the polypeptide sequence of IYO from the starting methionine until aa 430, 500, 959, and 978, respectively, as well as the complementary constructs IYO_500REV_, IYO_959REV_, and IYO_978REV_, which comprise the sequences from aa 500, 959, or 978 until aa 1,465 (**Figure 1**). Translational fusions to the N-terminal end of the GFP protein under the control of the *35S* promoter were transiently expressed in leaves of *N. benthamiana*, and their subcellular localization was analyzed by confocal microscopy. The fusion of the full-length IYO protein to GFP showed a homogeneous distribution of fluorescence in the nucleoplasm of epidermal cells, with negligible accumulation in the cytosol (**Figure 1**). Truncated constructs containing the RPAP1_N and RPAP1_C domains (IYO_ATG430_-GFP, IYO_ATG500_-GFP, IYO_ATG959_-GFP and IYO_ATG978_-GFP) also had an exclusively nuclear location, but with higher concentration in particular subnuclear structures (**Figure 1**). Subnuclear bodies have been related to transcription, processing, and compartmentalization of RNA (Lamond and Spector, 2003; Stanek and Fox, 2017), a role that would be consistent with the association of IYO with Pol II. All these constructs were excluded from the nucleolus, the site of ribosomal RNA transcription by RNA Pol I. The truncated versions of the C-terminal end (IYO_500REV_-GFP, IYO_959REV_-GFP and IYO_978REV_-GFP) presented mainly a cytoplasmic localization, although some fluorescence was observed in the nucleus (**Figure 1**). These results suggest that IYO may have two NLSs, a dominant one in the N-terminus of the protein (within the first 430 aa) and a weaker one in the C-terminus (between aa 979 and 1,465). Moreover, they indicate that constructs lacking the C-terminal part of the protein show preferential recruitment to subnuclear bodies.

**Figure 2:**
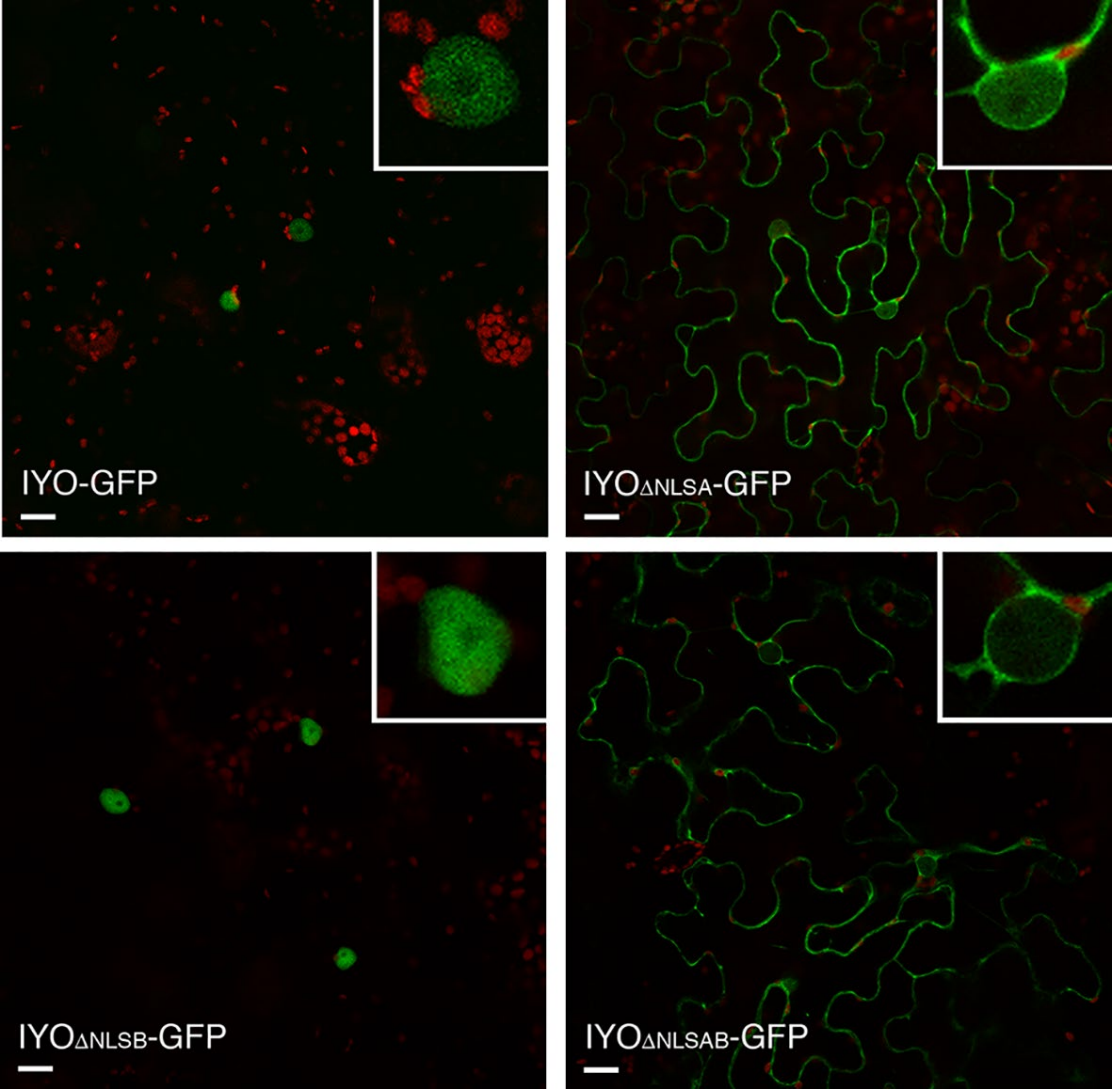
NLSs are responsible for IYO nuclear import. Single confocal sections of *N. benthamiana* leaf epidermal cells transiently transformed with IYO, IYO_ΔNLSA_, IYO_ΔNLSB_, and IYO_ΔNLSAB_ constructs fused to GFP. Scale bar: 20 µm.

Analysis of the IYO sequence with NLS prediction software (Kosugi et al., 2009; Nguyen Ba et al., 2009) revealed two putative NLSs in the protein (**Supplementary Figure 1**). One is a predicted monopartite NLS located between positions 254 and 259 (NLSA) of the IYO protein. The other is a bipartite NLS with two basic domains separated by a spacer sequence of 10 aa located between positions 1,401 and 1,417 (NLSB). To determine the role of the predicted motifs, we deleted them individually or together and analyzed the effect on the localization of GFP fusion constructs transiently expressed in *Nicotiana* (**Figure 2**). The protein mutated in the NLSA domain (IYO_ΔNLSA_-GFP) accumulated primarily in the cytosol and only weakly inside the nucleus, differing clearly from the exclusive nuclear localization of the wild-type IYO-GFP fusion protein. In contrast, the protein with the NLSB mutated (IYO_ΔNLSB_-GFP) accumulated inside the nuclei of leaves, similarly to wild-type IYO-GFP. Importantly, when both the NLSA and NLSB were mutated (IYO_ΔNLSAB_-GFP), the fusion protein was found in the cytosol (**Figure 2**). Moreover, nuclear accumulation of IYO_ΔNLSAB_-GFP was not observed even when nuclear export was inhibited by treatment with leptomycin B (see **Figure 8**), implying that nuclear import of the protein was blocked. These results are consistent with the observations made with the truncated constructs and support that IYO contains two functional NLS motifs involved in nuclear import, NLSA and NLSB.

**Figure 3:**
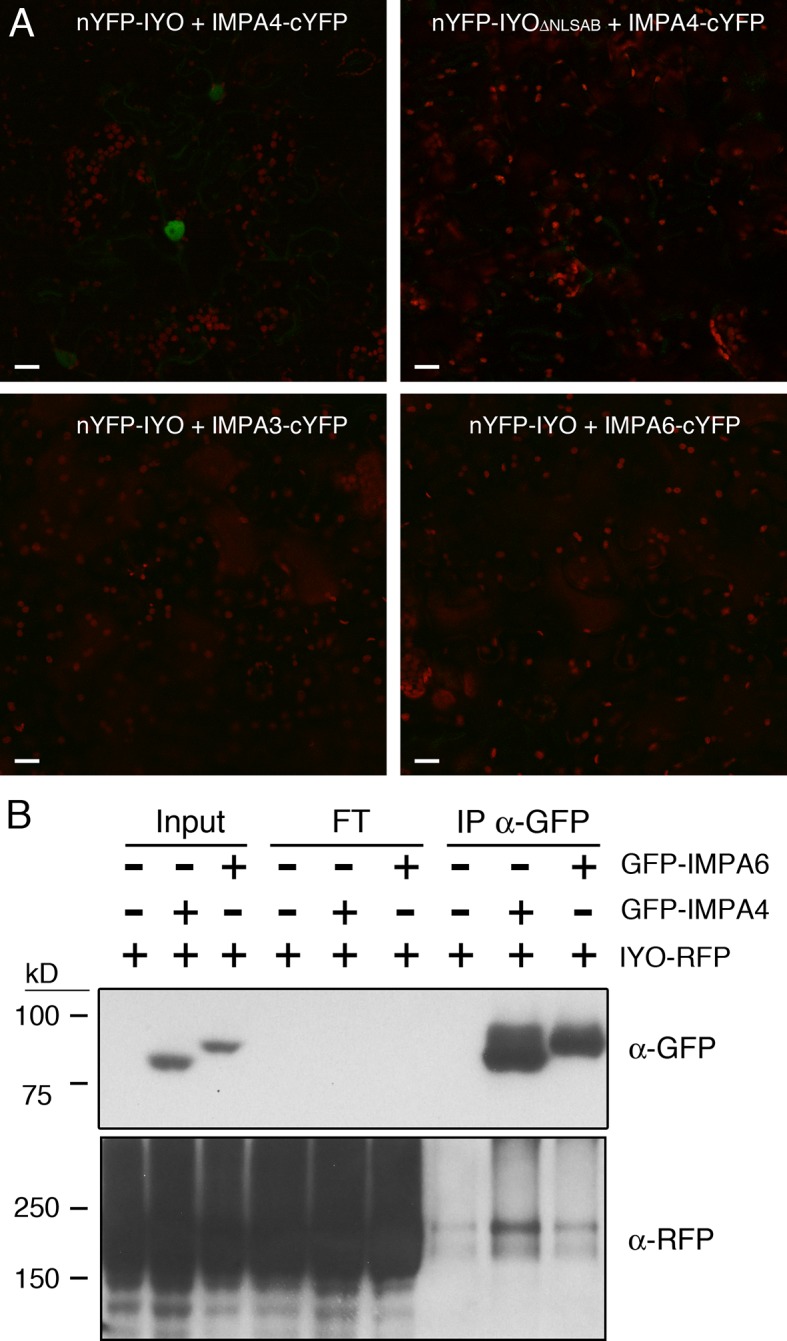
IYO interacts specifically with IMPA4 through its NLSs. **(A)** BiFC assay with the N-terminal half of YFP fused to IYO (nYFP-IYO) or to IYO_ΔNLSAB_ (nYFP-IYO_ΔNLSAB_) and the C-terminal half of YFP fused to IMPA3 (IMPA3-cYFP), IMPA4 (IMPA4-cYFP), or IMPA6 (IMPA6-cYFP). YFP signal is shown in green; chloroplasts signal in red. Scale bars: 20 µm. **(B)** Samples from *Nicotiana benthamiana* leaves infiltrated (+) or not (−) with IYO-RFP, GFP-IMPA4, and GFP-IMPA6 were immunoprecipitated with GFP-Trap agarose beads. Aliquots from the input, flow through, and immunoprecipitated fractions were analyzed by immunoblot with anti-GFP (upper panel) and anti-RFP (lower panels) antibodies.

NLSs are recognized by the importin-α subunits from nuclear transport receptors (Lange et al., 2007). Interestingly, in previous experiments to identify by mass-spectrometry proteins co-immunopurifying with a functional IYO-GFP protein (Muñoz et al., 2017), we found the importin-α protein IMPA4 with an enrichment ratio of 6.9 in samples purified from IYO-GFP plants relative to samples purified from wild-type plants. *IMPA4* is one of nine genes encoding importin-α proteins that are present in the *Arabidopsis* genome. Three of those genes, *IMPA3*, *IMP4*, and *IMPA6*, share with *IYO* a common longitudinal pattern of expression in the developing root (longitudinal pattern 8, Brady et al., 2007), suggesting possible functional relation among them. As expected for nuclear import factors, IMPA3, IMPA4, and IMPA6 were distributed between the nucleus and the cytosol (**Supplementary Figure 2**). To test the interaction of IYO with these importin-α proteins, we performed bimolecular fluorescence complementation (BiFC) analyses. YFP reconstitution was observed in the nucleus when nYFP-IYO and IMPA4-cYFP were co-expressed (**Figure 3A**), consistent with their co-purification by immunoprecipitation. Moreover, deletion of the NLSA and NLSB in IYO prevented this interaction (**Figure 3A**), suggesting that IMPA4 binds IYO through those NLSs. Interestingly, we did not observe fluorescence reconstitution when IMPA3-cYFP or IMPA6-cYFP were co-expressed with nYFP-IYO, which indicates specificity of IMPA4 for the IYO NLSs. To gain further support for this specificity, we tested for co-immunoprecipitation of IYO-RFP with GFP-IMPA4 and GFP-IMPA6 from extracts of *N. benthamiana* plants transformed with the constructs. We consistently observed co-precipitation of IYO-RFP with GFP-IMPA4 purified with anti-GFP antibodies, which was clearly above the background binding to the beads observed in extracts from plants that only expressed IYO-RFP (**Figure 3B**). In contrast, no enrichment over background was observed when GFP-IMPA6 was immunoprecipitated. Together, these results support that NLSA and NLSB are functional NLSs for IYO import into the nucleus that are possibly bound by IMPA4 *in vivo*.

**Figure 4:**
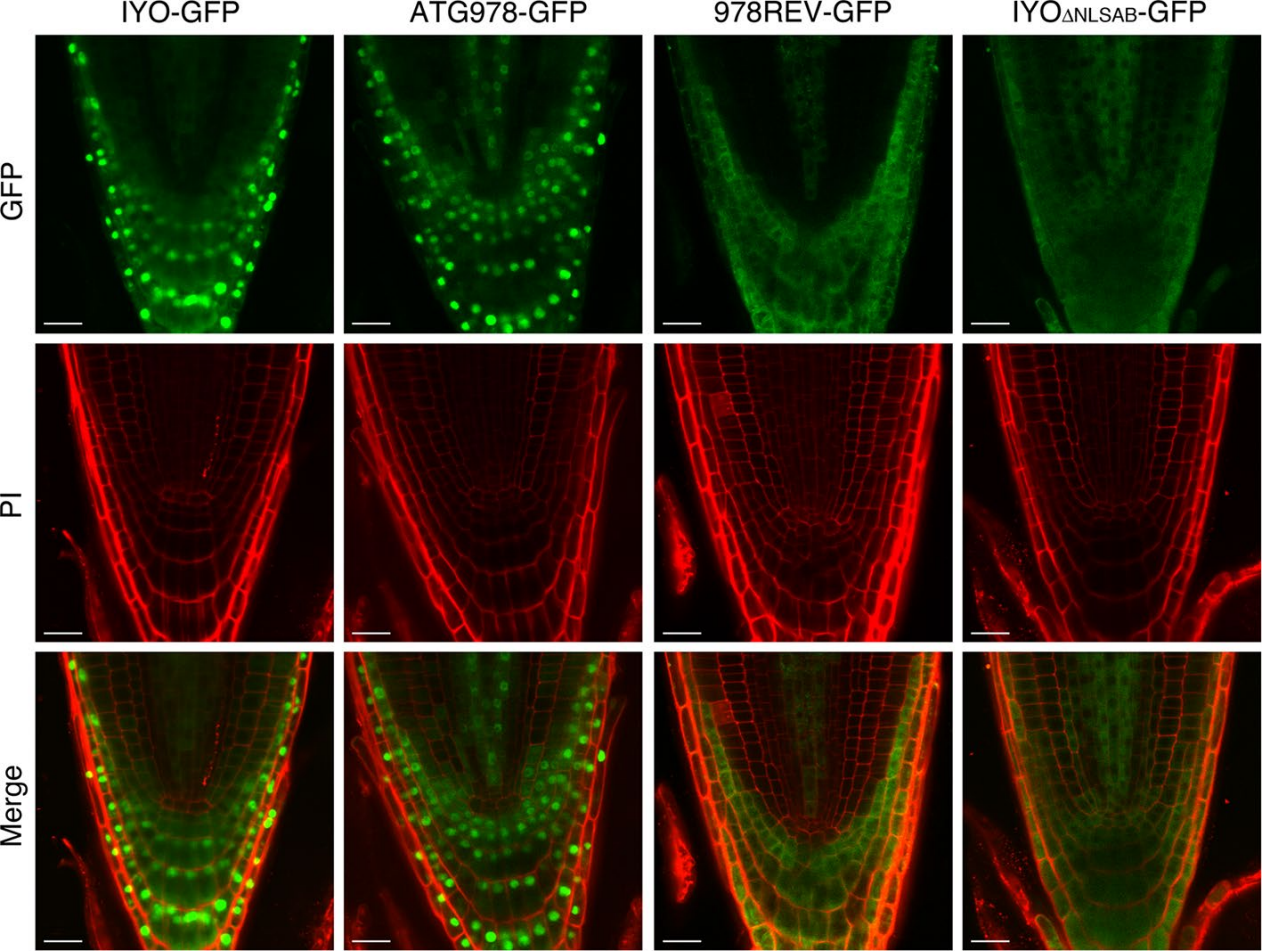
Nuclear IYO accumulation in differentiating root cells requires the NLSs. Single confocal sections of root tips from *35S:IYO-GFP* (IYO-GFP), *35S:IYO_ATG978_-GFP* (ATG978-GFP), *35S:IYO_978REV_-GFP* (978REV-GFP), and *35S:IYO*_ΔNLSAB_ *-GFP* (IYO_ΔNLSAB_-GFP) transgenic lines. GFP signal (upper panels), propidium iodide signal (middle panels), and a merge of both signals (lower panels) are shown. Scale bar: 20 µm.

To confirm the results obtained in *Nicotiana* and verify in *Arabidopsis*, the role of these NLSs in IYO nuclear import, we generated stably transformed lines expressing IYO-GFP, IYO_ATG978_-GFP, IYO_978REV_-GFP, and IYO_ΔNLSAB_-GFP and visualized the fluorescence distribution in the root apical meristem (**Figure 4**). As previously described (Sanmartin et al., 2011), IYO-GFP accumulated inside the nucleus in differentiated cells of the root cap, but not in undifferentiated cells of the root meristem, consistent with the notion that nuclear IYO activates differentiation. IYO_ATG978_-GFP was nuclear localized in differentiated root tip cells, but, interestingly, also in initial cells of the columella, of the epidermis/lateral root cap and of the cortex/endodermis, and in transit-amplifying cells of the vasculature, suggesting that sequences in the C-terminal end of the protein (aa 978–1,465) direct nuclear export of IYO from non-differentiating initial and transit amplifying cells. In the IYO_978REV_-GFP, GFP fluorescence accumulated in the cytosol of root cells. Moreover, in the IYO_ΔNLSAB_-GFP lines, the nuclei were clearly distinguished by the complete lack of GFP labeling (**Figure 4**). These results suggest that in *Arabidopsis*, NLSA, and to a lesser extent NLSB, direct nuclear import of IYO in differentiating cells of the root apical meristem. Together, the results obtained in *Nicotiana* and *Arabidopsis* strongly support that NLSA and NLSB are *bona fide* NLSs recognized by importin-α subunits for transport into the nucleus.

### Dissecting the Interaction of IYO With GPN1, a Factor Involved in Selective Nuclear Transport

**Figure 5:**
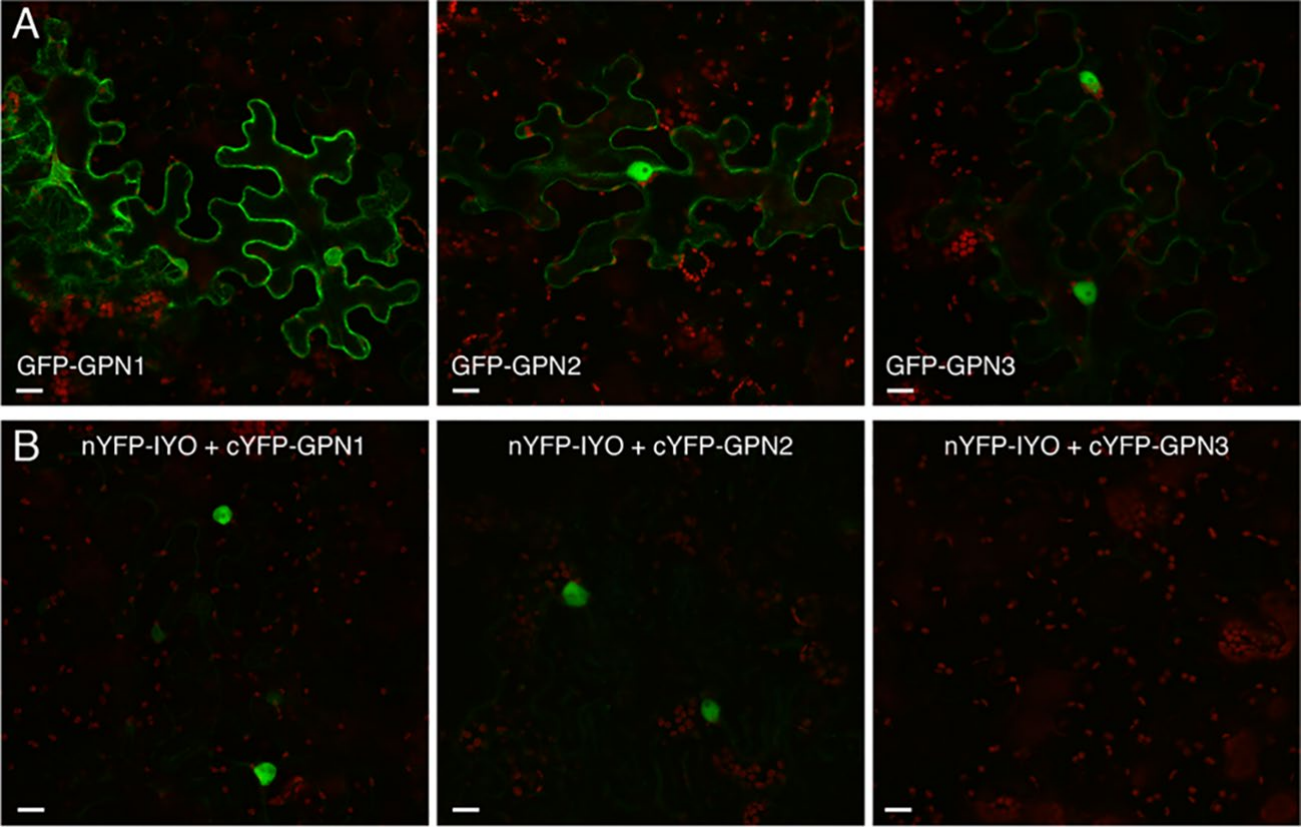
IYO interacts with specific GPNs. **(A)** Confocal images of *N. benthamiana* leaf epidermal cells transiently transformed with GFP-GPN1, GFP-GPN2, and GFP-GPN3 proteins. **(B)** BiFC assays between the N-terminal half of YFP fused to IYO (nYFP-IYO) and the C-terminal half of YFP fused to GPN1 (cYFP-GPN1), GPN2 (cYFP-GPN2), or GPN3 (cYFP-GPN3). Green: GFP/YFP fluorescence signal; red: chloroplasts. Scale bar: 20 µm.

**Figure 6:**
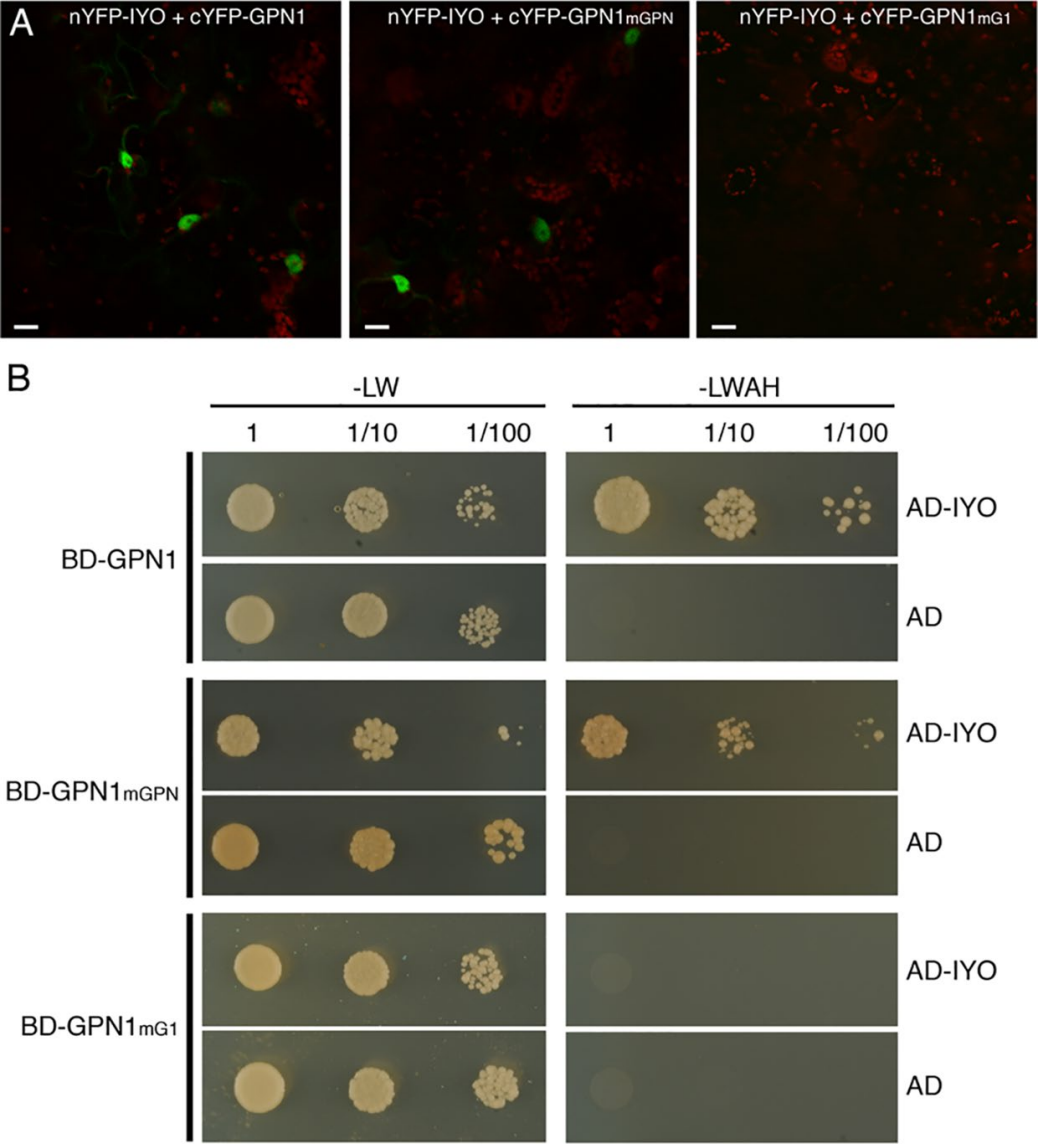
The nucleotide-bound form of GPN1 interacts with IYO. **(A)** BiFC assays between with nYFP-IYO and cYFP-GPN1_mGPN_ or cYFP-GPN1_mG1_. Green: YFP signal; red: chloroplasts signal. Scale bar: 20 µm. **(B)** Yeast cells cotransformed with BD-GPN1, BD-GPN1_mGPN_, or BD-GPN1_mG1_ (preys) and AD-IYO (bait) or empty pGAD vector (AD) were selected and subsequently grown on yeast synthetic dropout lacking Leu and Trp (−LW) as a transformation control or on selective media lacking Ade, His, Leu, and Trp (−LWAH) to test protein interactions.

**Figure 7:**
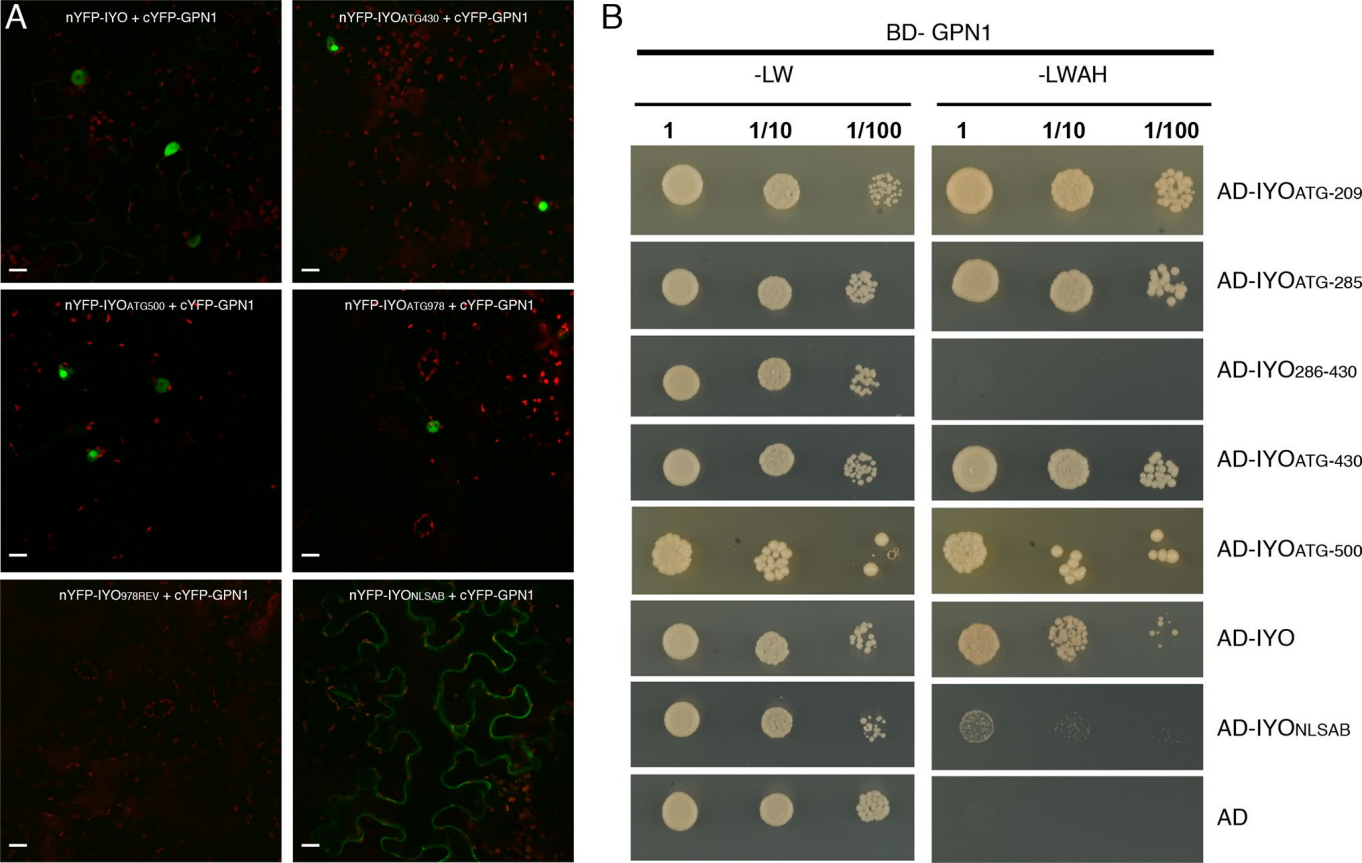
The N-terminal end of IYO is involved in GPN1 binding. **(A)** BiFC assays between nYFP-IYO or truncated versions nYFP-IYO_ATG430_, nYFP-IYO_ATG500_, nYFP-IYO_ATG978_, nYFP-IYO_978REV_, nYFP-IYO_NLSAB_, and cYFP-GPN1. Green: YFP signal; red: chloroplasts. Scale bar: 20 µm. **(B)** Y2H assay with BD-GPN1 (prey) and IYO truncated versions (AD-IYO_ATG209_, AD-IYO_ATG285_, AD-IYO_286-430_, AD-IYO_ATG430_, AD-IYO_ATG500_), AD-IYO and AD-IYO_NLSAB_ (baits), or empty pGAD vector (AD) were selected and subsequently grown on yeast synthetic dropout lacking Leu and Trp (−LW) as a transformation control or on selective media lacking Ade, His, Leu, and Trp (−LWAH) to test protein interactions.

**Figure 8:**
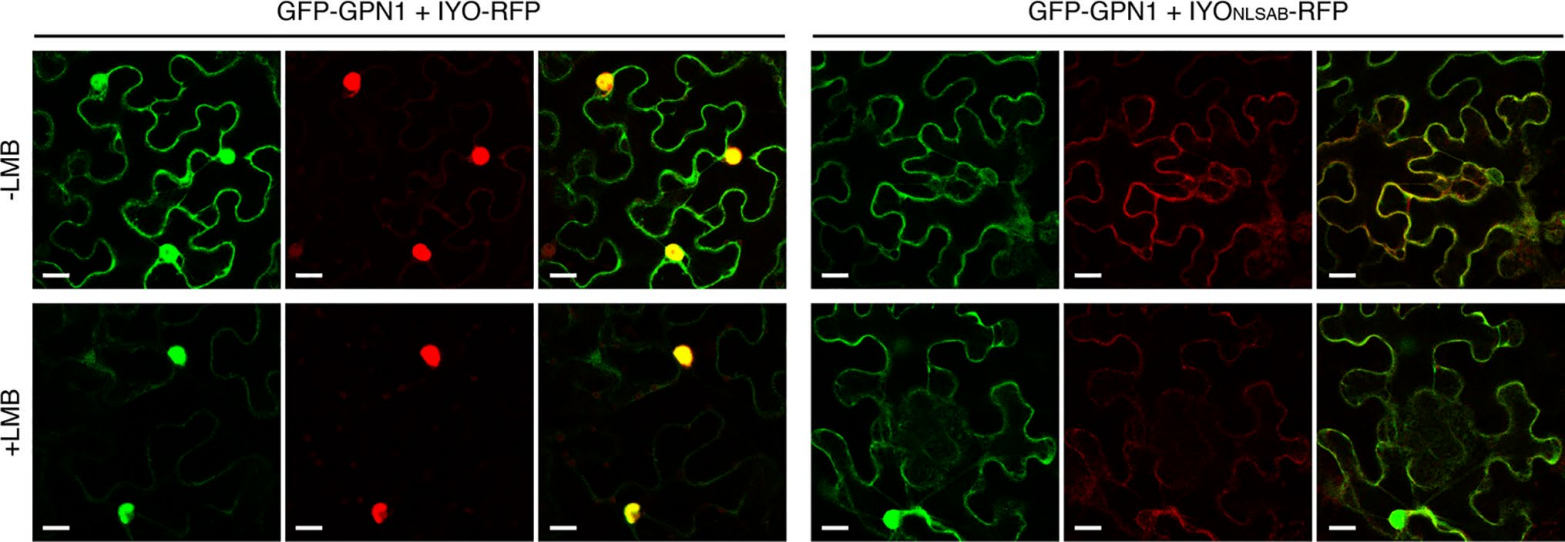
IYO and GPN1 are co-transported into the nucleus. Confocal images of *Nicotiana benthamiana* leaf epidermal cells transiently transformed with GFP-GPN1 and IYO-RFP or IYO_ΔNLSAB_-RFP in the absence (upper panels, −LMB) or presence (lower panels, +LMB) of leptomycin. GFP fluorescence signal (green channel), RFP fluorescence signal (red channel), and an overlay of both signals are shown. Scale bar: 20 µm.

Previous findings revealed that IYO interacts with GPN1 and GPN2 (Muñoz et al., 2017; Li et al., 2018), two members of the GPN family of GTPases that have been linked to selective nuclear transport of Pol II (Forget et al., 2010; Carre and Shiekhattar, 2011; Staresincic et al., 2011; Minaker et al., 2013; Zeng et al., 2018). In *Arabidopsis*, there are three GPNs, GPN1 to GPN3. When expressed in *Nicotiana*, GFP-GPN1 and GFP-GPN2 localize in the cytosol and the nucleus, whereas GFP-GPN3 is eminently nuclear (**Figure 5A**). Interestingly, IYO interacts in BiFC assays with GPN1 and GPN2 (Muñoz et al., 2017), but not with GPN3 (**Figure 5B**), suggesting binding specificity for certain GPN isoforms. Indeed, specific binding of IYO to GPN1 and not to GPN3 was confirmed by yeast two hybrid (Y2H) assays (**Supplementary Figure 3**). To characterize which domains are involved in the interaction between IYO and GPN proteins, we focused on GPN1. Alanine substitutions in the GPN loop (GPN to AAA) or in the G1 motif (GSGK to AAAA) of human RPAP4/GPN1 create dominant versions that interfere with Pol II nuclear accumulation when expressed in HeLa cells (Forget et al., 2010; Carre and Shiekhattar, 2011). We introduced equivalent mutations in *Arabidopsis* GPN1 and investigated their effect on the interaction and localization of IYO by Y2H and BiFC assays. These analyses showed that GPN1 with alanine substitutions in the GPN motif (GPN1_mGPN_) retained the binding to IYO (**Figures 6A–B**, **Supplementary Figure 4**), indicating that this motif is not involved in the interaction with IYO. Indeed, GPN3, which has the conserved GPN motif, does not bind IYO (**Figure 5B**; **Supplementary Figure 3**). Interestingly, the reconstituted BiFC complex between GPN1_mGPN_ and IYO was localized in the nucleus (**Figure 6A**), suggesting that expression of this GPN1 mutant form does not interfere with IYO nuclear accumulation, contrary to what happens to Pol II nuclear import in HeLa cells. Remarkably, no interaction with IYO was observed when the G1 motif, required for nucleotide-binding, was mutated in GPN1 (**Figures 6A–B**), indicating that IYO associates specifically with the nucleotide-bound form. We then searched for domains in IYO that may be required for interaction with GPN1. For this, we first analyzed by BiFC the interaction of GPN1 with a set of truncated IYO constructs. These analyses show that the GPN1-binding domain is localized in the first 500 aa of the IYO protein (**Figure 7A**). To corroborate these results and further delimit the GPN1-binding domain, we studied by yeast two-hybrid the interaction of GPN1 with an IYO deletion series (**Figure 7B**). These analyses established that the GPN-binding domain in IYO is localized in the first 209 aa of the protein. Moreover, deletion of NLSA and NLSB that abrogates the interaction with IMPA4 does not interfere with the binding to GPN1 (**Figure 7**). Interestingly, the BiFC GPN1/IYO_NLSAB_ complex was found in the cytosol, in contrast to the nuclear localization of the BiFC complex with the full-length IYO. These results suggest that GPN1 binds IYO in the cytoplasm and that import of the complex into the nucleus requires the NLSs from IYO. A caveat to this latter conclusion is that YFP reconstitution is thought to be irreversible, which could artificially alter the final subcellular distribution of the subunits of the complex. To exclude this possibility, we co-expressed GPN1 and IYO with or without the NLSs fused to different fluorophores that do not form irreversible complexes. Co-expression of GFP-GPN1 with IYO-RFP increased slightly the nuclear GFP levels relative to GFP-GPN1 expressed alone (compare **Figure 8** with **Figure 5A**). In contrast, co-expression of GFP-GPN1 with IYO_ΔNLSAB_-RFP resulted in an increased retention of GFP-GPN1 in the cytosol, which was more evident when nuclear export was inhibited by leptomycin B treatment (**Figure 8**). In leptomycin B-treated leaves, GFP-GPN1 co-expressed with IYO-RFP was found exclusively in the nucleus, whereas it was largely retained in the cytosol when co-expressed with IYO_ΔNLSAB_-RFP (**Figure 8**). These results support that IYO and GPN1 form a complex while still in the cytosol and are co-transported into the nucleus dependent on the IYO NLSs. However, GPN1mG1, which cannot interact with IYO, localizes partially to the nucleus and is almost entirely nuclear when export is inhibited with leptomycin B (**Supplementary Figure 5**), suggesting that there is also IYO-independent nuclear import of GPN1.

## Discussion

Despite the evidence that IYO/RPAP1 subcellular distribution is key for determining stem cell fate in plant and animals (Muñoz et al., 2017; Lynch et al., 2018), the domains responsible for translocation into the nucleus and the possible mechanisms involved in nuclear import were unknown. The work presented here reveals the primary role of NLSA in nuclear import of IYO. Removal of NLSA causes a very significant decrease in IYO nuclear levels. In contrast, elimination of just the bipartite NLSB domain does not significantly affect the nuclear levels of IYO, and its function in nuclear import is only revealed when the dominant NLSA domain is concomitantly mutated. Having identified these NLSs, a key objective for future studies will be to analyze how their mutation affects the *in vivo* activity of IYO, and in that way, establish in what compartment IYO exerts its pro-differentiation function.

Interestingly, the NLSs identified appear to be required also for nuclear import of GPN1, which would piggyback ride on IYO. This mechanism is reminiscent of the import of Pol II in yeast cells. Pol II subunits do not have NLSs, so the Pol II complex associates with the NLS-containing protein IWR1 for import into the yeast nucleus, a function that appears to be also conserved in human IWR1 (Czeko et al., 2011). Interestingly, IYO and GPN1 have been found in a Pol II subassembly complex that includes the *Arabidopsis* IWR1 homologue DMS4 (Li et al., 2018). It is thus plausible that the NLSs from IYO and DMS4 cooperate for import of a macro-complex formed by Pol II subunits and associated RPAP factors.

NLSA is a classical monopartite NLS located in the N-terminal end of the IYO protein between the conserved RPAP1_N and RPAP1_C domains. The NLSA motif itself shows sequence conservation and is predicted by NLS prediction algorithms, in IYO homologues from all embryophytes, suggesting that it plays a role in nuclear import of IYO proteins in all land plants. In contrast, the NLSB domain is only predicted in certain plants, such as *Vitis vinifera*, but it is absent in others like rice, consistent with its minor role in IYO nuclear import. Moreover, neither the NLSA nor the NLSB motifs are conserved outside the plant kingdom. *In silico* predictions suggest that the mouse RPAP1, which is also conditionally nuclear (Lynch et al., 2018), contains a monopartite NLS between the aa 70 and 80 of the protein. This putative NLS is upstream of the RPAP1_N domain and does not align with the NLSA motif from plant orthologues. The functionality of this NLS in nuclear import of RPAP1 remains to be tested.

IYO interacts with IMPA4, and this interaction is dependent on the presence of the NLSA and NLSB domains, indicating that IYO enters the nucleus through the canonical importin-α/importin-β transport pathway (Dingwall and Laskey, 1991). Interestingly, our results suggest differential affinity of the *Arabidopsis* importin-α isoforms for the IYO NLSs. *Saccharomyces cerevisiae* contains a single importin-α gene, compared to three in drosophila, seven in humans, and nine in *Arabidopsis*. The expansion of this gene family in *Arabidopsis* is consistent with functional diversification of the isoforms, possibly to accommodate a more complex regulation of nuclear transport. Such functional diversification could arise from differences in expression patterns or in cargo selectivity of the specific importin-α isoforms. There are prior examples of cargo specificity by importin-α isoforms in different organisms (Wirthmueller et al., 2013). For instance, the NLS of COP1 shows affinity for the rice importin-a1a and importin-a1b isoforms, but not for importin-a2 (Jiang et al., 2001) and the *Hyaloperonospora arabidopsidis* effector HaRxL106 is bound by *Arabidopsis* IMPA1, IMPA2, IMPA3, and IMPA4, but not by IMPA9 (Wirthmueller et al., 2015). Likewise, the phosphoinositide kinase PIP5K2 interacts with IMPA6 and IMPA9, but not with IMPA1-4 (Gerth et al., 2017). In addition, recent evidence indicates IMPA2 and IMPA4 have higher affinity than IMPA1, IMPA3, IMPA6, and IMPA9 for the NLS of PARP2 (Chen et al., 2018). Moreover, there is genetic evidence supporting functional divergence of IMPA4. A single mutant in IMPA4 impairs transformation by *Agrobacterium*, likely by interfering with nuclear import of the transfer DNA/protein complex, implying that IMPA4 serves specific functions and is not entirely redundant with other importin-α genes (Bhattacharjee et al., 2008). Our results extend these findings by showing that IMPA4, but not IMPA3 and IMPA6, associates with IYO in BiFC assays, suggesting differential binding specificity for this NLS cargo. Phylogenetic analyses show that *Arabidopsis* IMPA4 is closer in sequence to IMPA1 and IMPA2, forming a separate clade from IMPA3 and IMPA6 that form a separate cluster (Bhattacharjee et al., 2008; Wirthmueller et al., 2013), which could explain the differential affinity for the IYO NLSs. Further characterization using IYO as a model cargo could help determine how sequence divergence in the *Arabidopsis* importin-α family translates to changes in NLS binding affinity.

The interaction with GPNs may also prove relevant for IYO nucleo/cytoplasmic partitioning. GPNs are a family of GTPases related to the RAN nuclear transport factors that have been linked to selective nuclear transport of Pol II in humans and yeast (Forget et al., 2010; Calera et al., 2011; Carre and Shiekhattar, 2011; Staresincic et al., 2011; Minaker et al., 2013; Zeng et al., 2018). The interaction between IYO/RPAP1 and GPNs has been conserved throughout evolution in yeast, mammals, and plants (Boulon et al., 2010; Muñoz et al., 2017; Li et al., 2018; Zeng et al., 2018) and may occur through common domains. The *IYO/RPAP1* homologues in yeasts encode shorter proteins (439 aa in *Saccharomyces cerevisiae*) that correspond essentially to the N-terminal end of plant and mammalian IYO/RPAP1 proteins, including the conserved RPAP1_N and RPA1_C domains. Hence, interaction with GPN proteins should occur through a conserved domain present in this N-terminal end that was already present in the ancestral IYO/RPAP1 protein. Consistent with this, our results show that *Arabidopsis* IYO interacts with GPN1 through the first 209 aa of the protein. Sequence comparison of this region among land plants homologues distinguishes two separate domains. A first domain (aa 20–112) with significant sequence conservation (48% identity between *Arabidopsis* and rice and 39% between *Arabidopsis* and the liverwort *Marchantia polymorpha*) and a second domain (aa 113–206) with negligible sequence conservation (15% identity between *Arabidopsis* and rice and 6% between *Arabidopsis* and *Marchantia*). Interestingly, a different splicing pattern in *Brassica oleracea* and *Brassica rapa* produces in these species IYO proteins without aa 90–213 from *Arabidopsis* IYO, which is essentially the domain showing low sequence conservation across plant homologues. Considering that interaction with GPN proteins has been maintained throughout evolution in all eukaryotic kingdoms and it is likely to be essential for IYO function, we can infer then that it is maintained in *Brassica oleracea* and *Brassica rapa*. This implies that binding of IYO to GPNs most likely occurs through the first 89 aa of the protein. Within this region, the most conserved motif is an FPVARHRS sequence (aa 49–55) that is invariant in IYO homologues from all land plants and has a single aa substitution in the homologues from the algae *Chara braunii* and the fungi *Cylindrobasidium torrendii*. This high level of conservation suggests that it is an important motif for IYO function and a good candidate to mediate GPN binding.

## Data Availability

All datasets generated for this study are included in the manuscript/Supplementary Files.

## Author Contributions

RC, PK, MPG-G, AL, MS, and ER performed the experiments and analyzed the data. JJS-S, MS, and ER designed and supervised the experiments. MS and ER wrote the manuscript. All authors discussed the results and commented on the manuscript.

## Funding

This work was supported by the Spanish Ministry of Economy & Competitivity and FEDER funds (BIO2018-094257-B MICINN/FEDER to ER, MS and JJSS). RC and AL were recipient of a FPI scholarships from the Spanish Ministry of Economy and Competitivity. PK was recipient of an ERASMUS fellowship. We acknowledge support of the publication fee by the CSIC Open Access Publication Support Initiative through its Unit of Information Resources for Research (URICI).

## Conflict of Interest Statement

The authors declare that the research was conducted in the absence of any commercial or financial relationships that could be construed as a potential conflict of interest.

## Supplementary Material

Supplementary Table 1 Primers used in this work.

Supplementary Figure 1 Conserved domains in IYO. Multiple sequence alignment of several IYO predicted proteins was performed using Clustal Omega in the EMBL-EBI software. The conserved RPAP and RGG domains are underlined in black and blue respectively. Identified NLS domains in AtIYO in this work are shown in red. Proposed putative GPN1 binding motif in underlined in green. AtIYO: *Arabidopsis thaliana*; VvIYO: *Vitis vinifera*, OsIYO: *Oryza sativa*, PtIYO: *Populus trichocarpa*, PpIYO: *Physcomitrella patents*, MpIYO: *Marchantia polymorpha*.

Supplementary Figure 2 Subcellular distribution of Importin-α. Confocal images of *Nicotiana benthamiana* leaf epidermal cells transiently transformed with GFP-IMPA3, GFP-IMPA4 and GFP-IMPA6. GFP signal is shown in green; Chloroplast signal in red. Scale bar: 20 µm.

Supplementary Figure 3 Specificity of IYO interaction with GPNs. Yeast cells cotransformed with BD-GPN1 or BD-GPN3 (preys) and AD-IYO (bait) were selected and subsequently grown on yeast synthetic dropout lacking Leu and Trp (−LW) as a transformation control or on selective media lacking Ade, His, Leu, and Trp (−LWAH) to test protein interactions. A known positive control for BD-GPN3 was used to verify transformation.

Supplementary Figure 4 The G1 domain in GPN1 is required for binding to IYO. Yeast cells cotransformed with AD-GPN1, AD-GPN1_mGPN_ or AD-GPN1_mG1_ (preys) and BD-IYO (bait) were selected and subsequently grown on yeast synthetic dropout lacking Leu and Trp (−LW) as a transformation control or on selective media lacking Ade, His, Leu, and Trp (−LWAH) to test protein interactions.

Supplementary Figure 5 Confocal images of *Nicotiana benthamiana* leaf epidermal cells transiently transformed with GFP-GPN1_mG1_ and incubated in the absence (upper panels, −LMB) or presence (lower panels, +LMB) of 1 mM leptomycin for 2 h before imaging. GFP fluorescence signal (green channel) and an overlay with bright field images are shown. A maximum intensity z-stack projection of 8 sections is shown. Scale bar: 10 µm.
