# Supplementary material for: Identification of Domains and Factors Involved in MINIYO Nuclear Import
Source: Front Plant Sci. 2019 Sep 5;10:1044. doi: 10.3389/fpls.2019.01044 (PMC6748027; doi:10.3389/fpls.2019.01044)
Supplement: Supplementary Table 1 — Primers used in this work. [file Table_1.docx]

**Supplementary Table 1**. Primers used in this work.

| **Primer name** | **Sequences** | **Construct** |
| --- | --- | --- |
| IYOATG | GGGGACAAGTTTGTACAAAAAAGCAGGCTTGATGGAGCAAAGTAGCGGGAGAG | IYO full length and N-terminal constructs |
| IYO209 Up | GGGGACCACTTTGTACAAGAAAGCTGGGTAACTTTCAAGAGACGAAGACGC | IYO_ATG209_ construct |
| IYO285 Up | GGGGACCACTTTGTACAAGAAAGCTGGGTACACTTTAGGAGTGACAAAATG | IYO_ATG285_ construct |
| IYO286Dw | GGGGACAAGTTTGTACAAAAAAGCAGGCTTGATGGCAATACCGAAAGAAAAA | IYO_286-430_ construct |
| IYO430 Up | GGGGACCACTTTGTACAAGAAAGCTGGGTAAGGTTCCGGTCCAAGGGCAT | IYO_286-430_ and IYO_ATG-430_ constructs |
| IYO500 Up | GGGGACCACTTTGTACAAGAAAGCTGGGTACCACGGAGGAAGCCAAGATC | IYO_ATG500_ construct |
| IYO959 Up | GGGGACCACTTTGTACAAGAAAGCTGGGTATTTATGTAGCTCAATTGATTGCACGATG | IYO_ATG959_ construct |
| IYO978 Up | GGGGACCACTTTGTACAAGAAAGCTGGGTACCCACCACCGCTAGCTCCCC | IYO_ATG978_ construct |
| IYO500Dw | GGGGACAAGTTTGTACAAAAAAGCAGGCTTGATGGATCTTGGCTTCCTCCGTGG | IYO_500REV_ construct |
| IYO959Dw | GGGGACAAGTTTGTACAAAAAAGCAGGCTTGATGATCGTGCAATCAATTGAGCTACATAAA | IYO_959REV_ construct |
| IYO978Dw | GGGGACAAGTTTGTACAAAAAAGCAGGCTTGATGTTTTGGTCAACCAGAGTTCTG | IYO_978REV_ construct |
| IYOnostop | GGGGACCACTTTGTACAAGAAAGCTGGGTACCTTCTTCCACAGAGAGCGGCT | IYO full length and C-terminal constructs |
| NLS DEL 74 | TCTGTCCAGGGGGTTTCCATCACC | Deletion of IYO NLSA domain |
| NLS DEL 68 | CAATTTTGCCTCACCTCGTTTCTT | Deletion of IYO NLSA domain |
| NLS DEL 59 | GAGGGGATGATGTTAGATCTC | Deletion of IYO NLSB domain |
| NLS DEL 62 | TGATAAATCCCTGACAAGAGTCTT | Deletion of IYO NLSB domain |
| mutGPN-AAA For | AGCGGGTGGAATTCTCACTTCAC | GPN domain of GPN1 mutagenesis |
| mutGPN-AAA Rev | GCTGCCAGATTATACTGCTTCATAAC | GPN domain of GPN1 mutagenesis |
| mutG1-AAAA For | GCGGCGAGCTTTCTTCATCGCTTG | G1 domain of GPN1 mutagenesis |
| mutG1-AAAA Rev | TGCTGCTGCCATTCCAACAACGAT | G1 domain of GPN1 mutagenesis |
| GPN1 F | GGACAAGTTTGTACAAAAAAGCAGGCTCCATGGATCCTATGGAGTCGTC | GPN1 CDS |
| GPN1 R nostop | GGGGACCACTTTGTACAAGAAAGCTGGGTATAGGTAGTAATGCTTCGTCTCGTCGTCT | GPN1 CDS without stop codon |
| GPN2 GW For | GGGGACAAGTTTGTACAAAAAAGCAGGCTTGATGGTGTTTGGACAAGTAGTAATAGG | GPN2 CDS |
| GPN2 GW Rev nostop | GGGGACCACTTTGTACAAGAAAGCTGGGTAGTCTTGTATTTCCTCATCTTCCATGTAC | GPN2 CDS without stop codon |
| GPN3 GW For | GGGGACAAGTTTGTACAAAAAAGCAGGCTTGATGGGTTACGCCCAGCTAGTTATTGG | GPN3 CDS |
| GPN3 GW Rev nostop | GGGGACCACTTTGTACAAGAAAGCTGGGTATAGGTCAGGACCATCGTCACTAAAATC | GPN2 CDS without stop codon |
| ImpA3 GW For | GGGGACAAGTTTGTACAAAAAAGCAGGCTTGATGTCTCTCAGACCTAGCGCGAAGAC | Importin 3 CDS |
| ImpA3 GW Rev nostop | GGGGACCACTTTGTACAAGAAAGCTGGGTAAATAAAGTTGAATTGACCAGGAGGAAC | Importin 3 CDS without stop codon |
| ImpA4 GW For | GGGGACAAGTTTGTACAAAAAAGCAGGCTTGATGTCGCTGAGGCCGAGCACACGCGC | Importin 4 CDS |
| ImpA4 GW Rev nostop | GGGGACCACTTTGTACAAGAAAGCTGGGTAGGCAAATTTGAATCCACCAACGGGAG | Importin 4 CDS without stop codon |
| ImpA6 GW For | GGGGACAAGTTTGTACAAAAAAGCAGGCTTGATGTCTTACAAACCAAGCGCGAAGAC | Importin 6 CDS |
| ImpA6 GW Rev nostop | GGGGACCACTTTGTACAAGAAAGCTGGGTAACCAAAGTTGAATCCACCCGTAGGAG | Importin 6 CDS without stop codon |
