# Supplementary figures and images for: Identification of Domains and Factors Involved in MINIYO Nuclear Import
Source: Front Plant Sci. 2019 Sep 5;10:1044. doi: 10.3389/fpls.2019.01044 (PMC6748027; doi:10.3389/fpls.2019.01044)

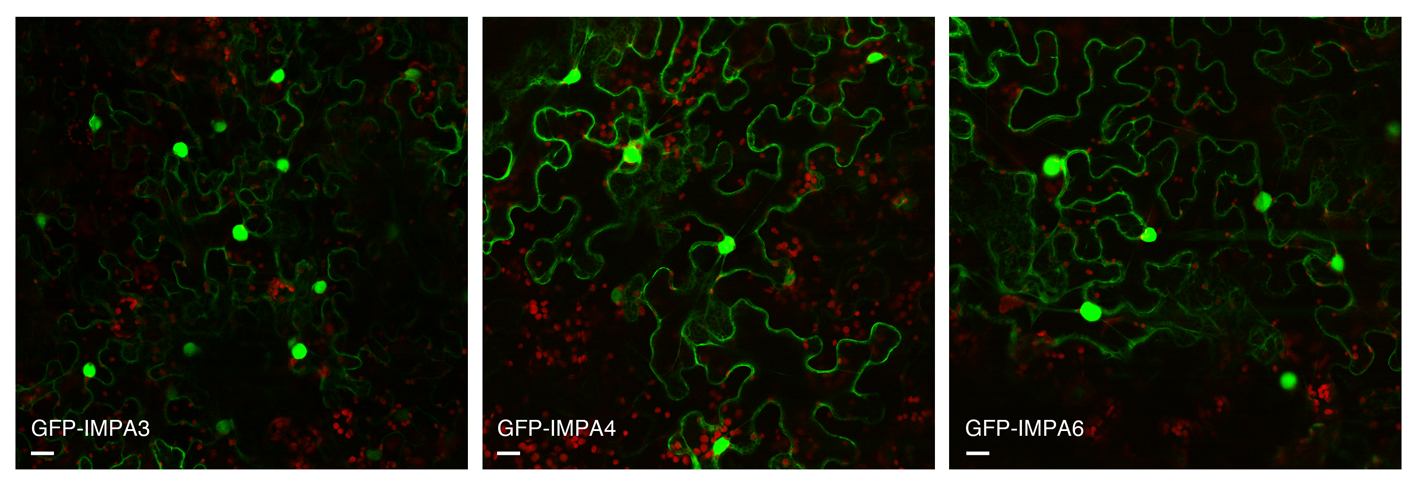

Supplement: Supplementary Figure 2 — Subcellular distribution of Importin-α. Confocal images of Nicotiana benthamiana leaf epidermal cells transiently transformed with GFP-IMPA3, GFP-IMPA4 and GFP-IMPA6. GFP signal is shown in green; Chloroplast signal in red. Scale bar: 20 µm. [file Image_2.tif]

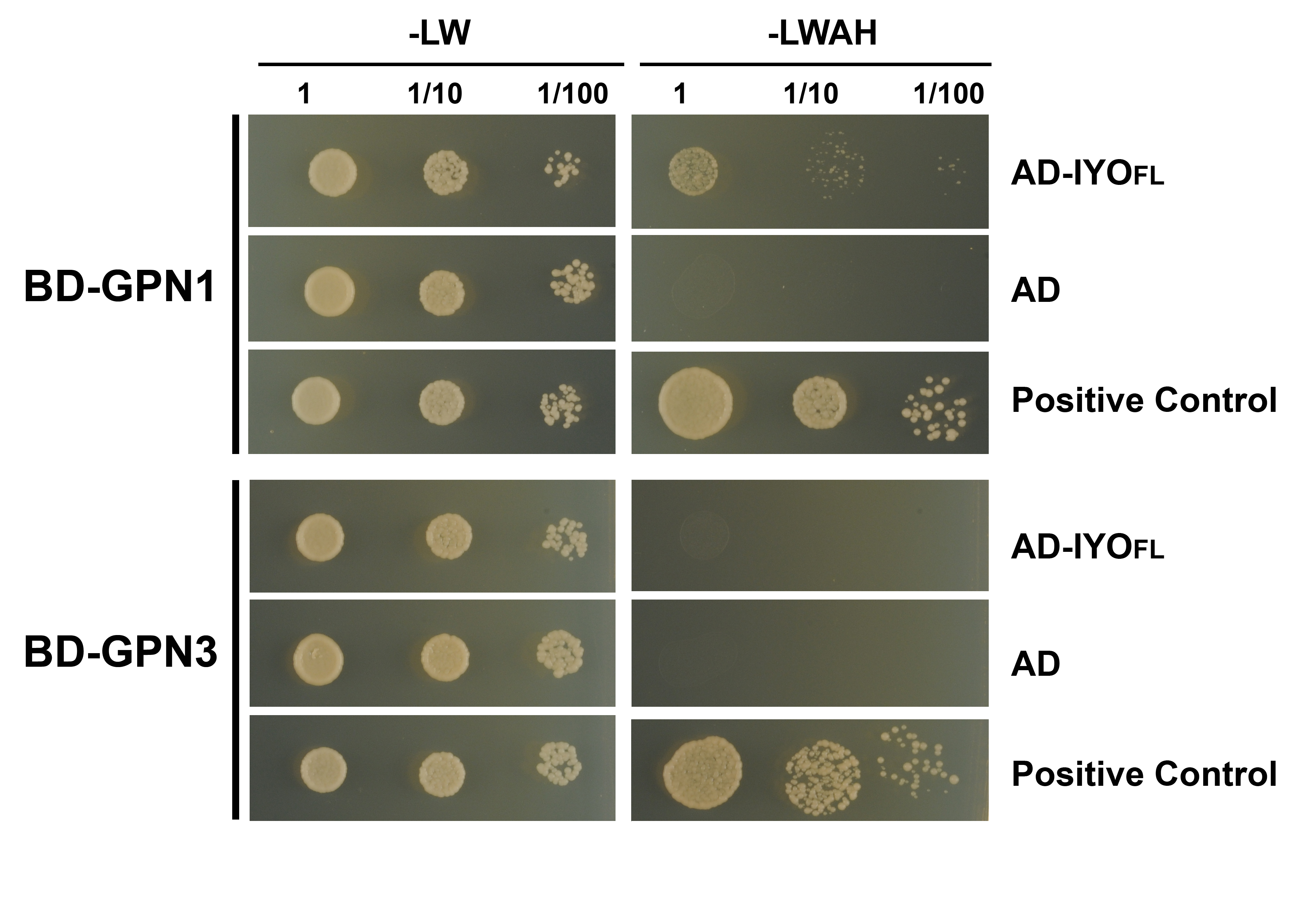

Supplement: Supplementary Figure 3 — Specificity of IYO interaction with GPNs. Yeast cells cotransformed with BD-GPN1 or BD-GPN3 (preys) and AD-IYO (bait) were selected and subsequently grown on yeast synthetic dropout lacking Leu and Trp (−LW) as a transformation control or on selective media lacking Ade, His, Leu, and Trp (−LWAH) to test protein interactions. A known positive control for BD-GPN3 was used to verify transformation. [file Image_3.jpeg]

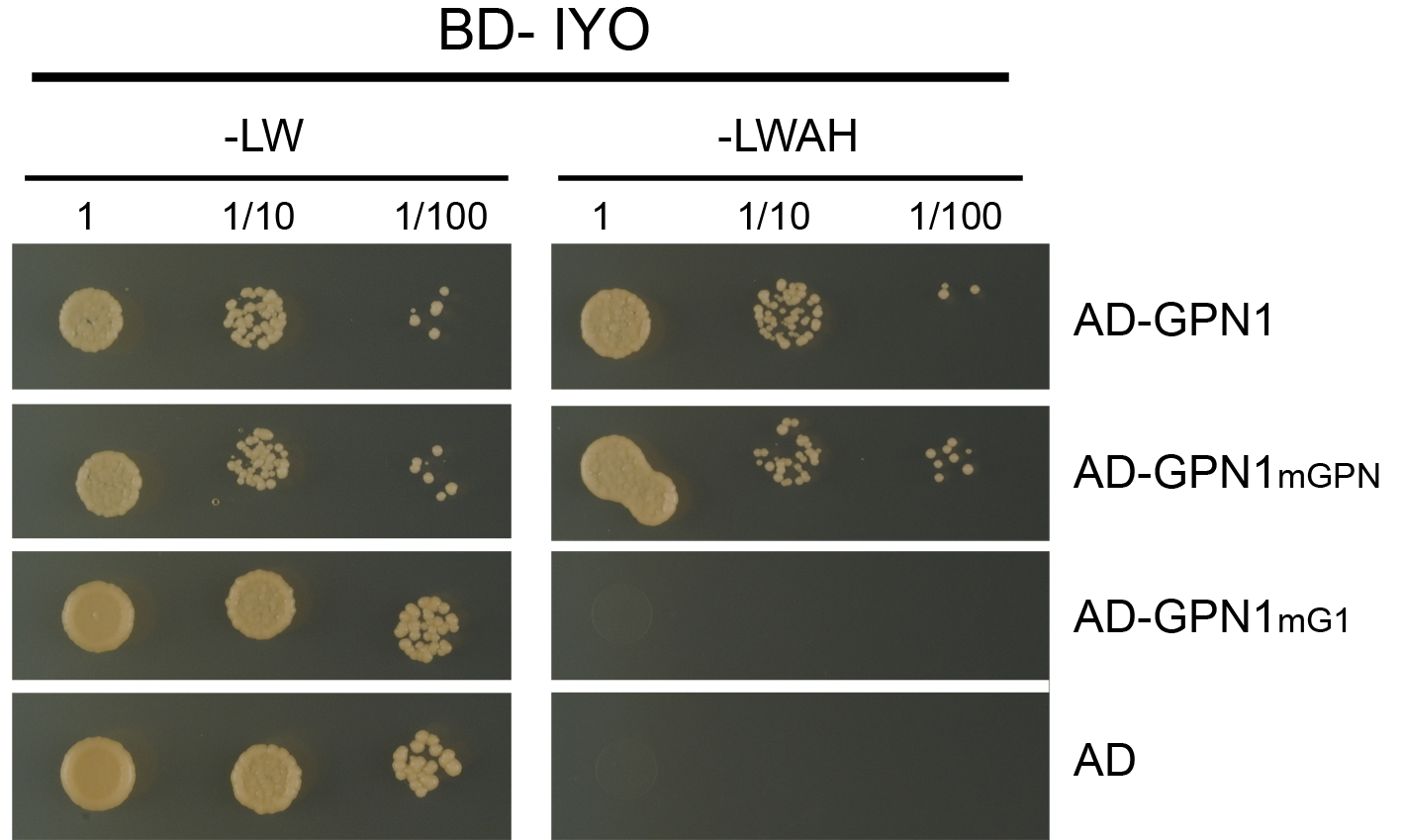

Supplement: Supplementary Figure 4 — The G1 domain in GPN1 is required for binding to IYO. Yeast cells cotransformed with AD-GPN1, AD-GPN1mGPN or AD-GPN1mG1 (preys) and BD-IYO (bait) were selected and subsequently grown on yeast synthetic dropout lacking Leu and Trp (−LW) as a transformation control or on selective media lacking Ade, His, Leu, and Trp (−LWAH) to test protein interactions. [file Image_4.tif]

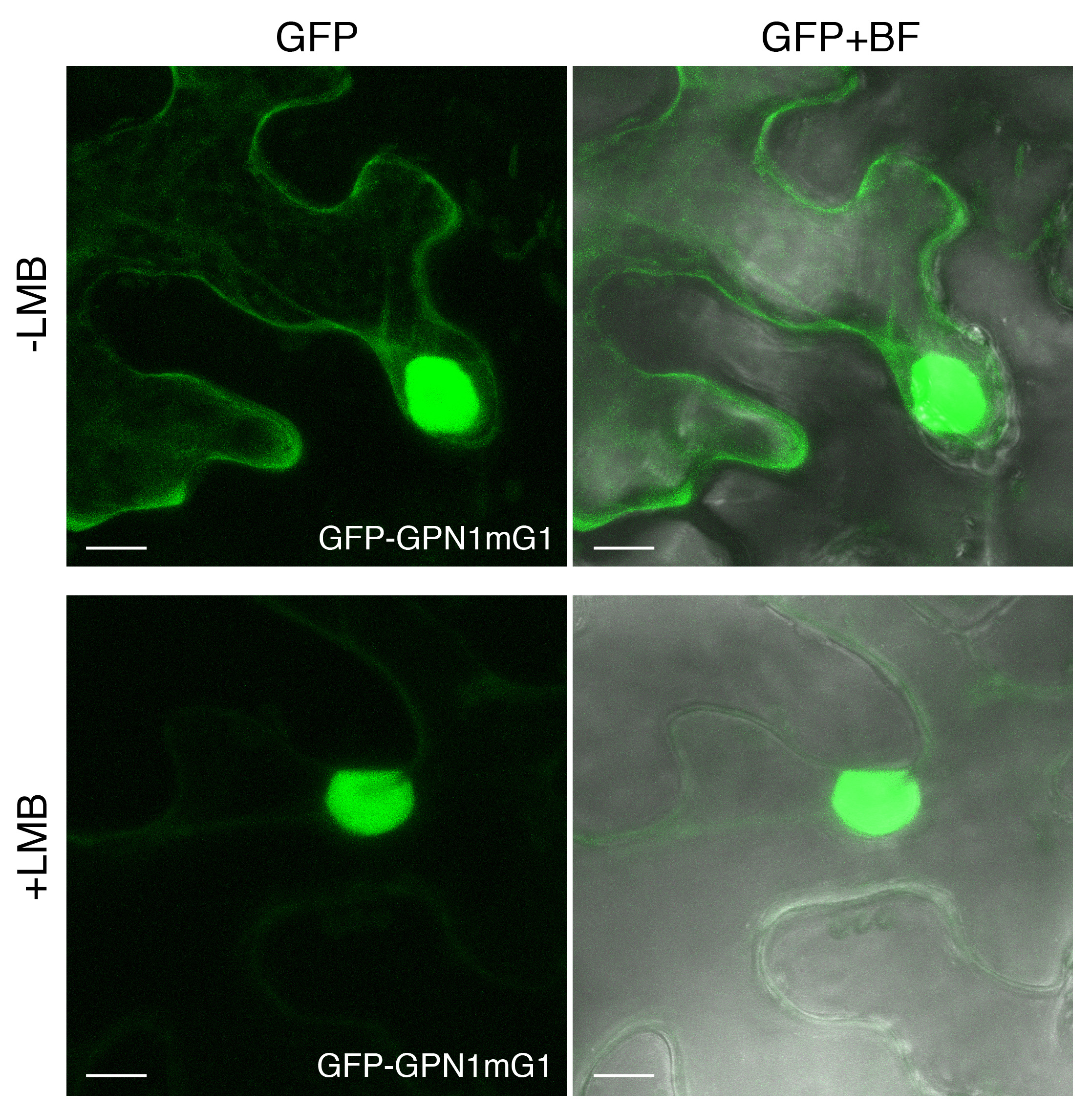

Supplement: Supplementary Figure 5 — Confocal images of Nicotiana benthamiana leaf epidermal cells transiently transformed with GFP-GPN1mG1 and incubated in the absence (upper panels, −LMB) or presence (lower panels, +LMB) of 1 mM leptomycin for 2 h before imaging. GFP fluorescence signal (green channel) and an overlay with bright field images are shown. A maximum intensity z-stack projection of 8 sections is shown. Scale bar: 10 µm. [file Image_5.jpeg]
