# Supplementary material for: Identification of Domains and Factors Involved in MINIYO Nuclear Import
Source: Front Plant Sci. 2019 Sep 5;10:1044. doi: 10.3389/fpls.2019.01044 (PMC6748027; doi:10.3389/fpls.2019.01044)
Supplement: Supplementary Figure 1 — Conserved domains in IYO. Multiple sequence alignment of several IYO predicted proteins was performed using Clustal Omega in the EMBL-EBI software. The conserved RPAP and RGG domains are underlined in black and blue respectively. Identified NLS domains in AtIYO in this work are shown in red. Proposed putative GPN1 binding motif in underlined in green. AtIYO: Arabidopsis thaliana; VvIYO: Vitis vinifera, OsIYO: Oryza sativa, PtIYO: Populus trichocarpa, PpIYO: Physcomitrella patents, MpIYO: Marchantia polymorpha. [file Image_1.pdf]

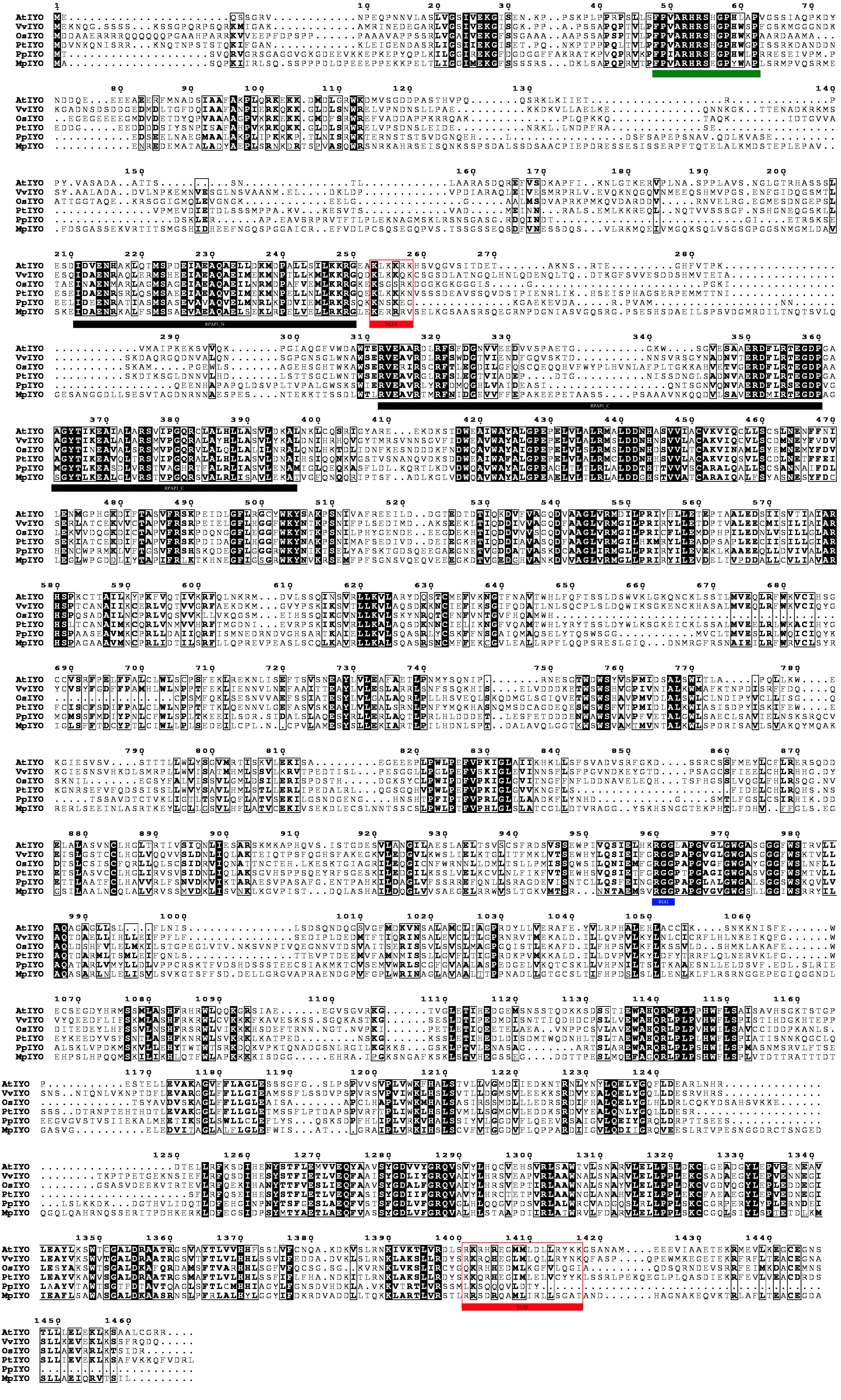
AtIYO
VvIYO
OsIYO
PtIYO
PpIYO
MpIYO
RPAP1_N
NLSA
RPAP1_C
RGG
NLSB
